# Outcomes of transsphenoidal surgery for pituitary adenomas in Spain: a retrospective multicenter study

**DOI:** 10.3389/fendo.2025.1529418

**Published:** 2025-02-21

**Authors:** Miguel Paja, Alfonso Soto, Felicia A. Hanzu, Fernando Guerrero-Pérez, Rosa Cámara, Dolores Moure, Ángeles Gálvez, Andreu Simó-Servat, Rocío Villar-Taibo, María Calatayud, Almudena Vicente, Jose M. Recio-Córdova, Guillermo Serra, Patricia Martín Rojas-Marcos, Paola Parra-Ramírez, Marta Araujo-Castro, Soledad Librizzi, Ana Irigaray, Dolores Ollero, Silvia Aznar, Fernando Muñoz, Anna Aulinas, Laura González-Fernández, Rogelio García-Centeno, Nerea Egaña, Tomás González-Vidal, Edelmiro Menéndez, Ana M. Delgado, Javier Abarca, Johana Sottile, Antonio M. Picó, Cristina Novo, Isabel Ortiz, Carmen Tenorio, Ricardo de León, Pedro de Pablos-Velasco, Cristina Crespo, David Peñalver, Gonzalo Díaz-Soto, Manel Puig-Domingo, Betina Biagetti

**Affiliations:** ^1^ Departament of Endocrinology and Nutrition, Hospital Universitario de Basurto, Bilbao, Spain; ^2^ Medicine Department, University of the Basque Country UPV/EHU, Bilbao, Spain; ^3^ Endocrinology and Nutrition Department, Hospital Universitario Virgen del Rocío, Sevilla, Spain; ^4^ Endocrinology and Nutrition Department, Hospital Clinic, IDIPAS, Barcelona, Spain; ^5^ Department of Endocrinology and Nutrition, Hospital Universitari de Bellvitge, Barcelona, Spain; ^6^ Departament of Endocrinology and Nutrition, La Fe University Hospital, Valencia, Spain; ^7^ Endocrinology and Nutrition Department, Hospital Universitario Cruces, Barakaldo, Bizkaia, Spain; ^8^ Department of Endocrinology and Nutrition, Hospital Universitario Reina Sofía, Córdoba, Spain; ^9^ Department of Endocrinology and Nutrition, Hospital Universitario Mutua de Terrassa, Terrassa, Spain; ^10^ Department of Endocrinology and Nutrition, Hospital Universitario Santiago de Compostela, Santiago de Compostela, Spain; ^11^ Department of Endocrinology and Nutrition, Hospital Universitario 12 de Octubre, Madrid, Spain; ^12^ Endocrinology and Nutrition Department, Hospital Universitario de Toledo, Toledo, Spain; ^13^ Endocrinology and Nutrition Department, Hospital Universitario de Salamanca, Salamanca, Spain; ^14^ Endocrinology and Nutrition Department, Hospital Universitario Son Espases, Mallorca, Spain; ^15^ Endocrinology and Nutrition Department, Hospital Universitario La Paz, Madrid, Spain; ^16^ Endocrinology and Nutrition Department, Hospital Universitario Ramón y Cajal, Madrid, Spain; ^17^ Department of Endocrinology and Nutrition, Hospital Universitario de Navarra, Pamplona, Spain; ^18^ Endocrinology and Nutrition Department, Hospital Universitario de Albacete, Albacete, Spain; ^19^ Neurosurgery Department, Hospital Sant Pau, Barcelona, Spain; ^20^ Endocrinology and Nutrition Department, Hospital Sant Pau, Barcelona, Spain; ^21^ Endocrinology and Nutrition Department, Hospital Universitario Gregorio Marañón, Madrid, Spain; ^22^ Endocrinology and Nutrition Department, Hospital Universitario Donostia, Donostia, Spain; ^23^ Department of Endocrinology and Nutrition, Hospital Central de Asturias, Oviedo, Spain; ^24^ Endocrinology and Nutrition Department, Hospital Universitario de Burgos, Burgos, Spain; ^25^ Department of Neurosurgery, Hospital General Universitario de Alicante, Alicante, Spain; ^26^ Department of Endocrinology, Hospital General Universitario de Alicante, Alicante, Spain; ^27^ Department of Endocrinology and Nutrition, Hospital Universitario de Granada, Granada, Spain; ^28^ Department of Neurosurgery, Hospital Universitario de Granada, Granada, Spain; ^29^ Department of Endocrinology and Nutrition, Hospital Dr Negŕın, Las Palmas de Gran Canaria, Las Palmas de Gran Canaria, Spain; ^30^ Department of Endocrinology and Nutrition, Hospital Clínico Universitario de Valladolid, Valladolid, Spain; ^31^ Department of Endocrinology and Nutrition, Hospital German Trías i Pujol, Badalona, Spain; ^32^ Department of Endocrinology and Nutrition, Hospital Universitario Vall d’Hebrón, CIBERER U747 (ISCIII), Barcelona, Spain

**Keywords:** transsphenoidal surgery, pituitary adenoma, surgical complications, success rate, surgical experience, surgical specialization

## Abstract

**Background:**

The outcomes of transsphenoidal surgery (TSS) for pituitary adenoma (PA) depend on many factors, including the availability of an expert team and the volume of surgeries performed. Data on the outcomes of TSS for PA are scarce in our country. TESSPAIN evaluates TSS outcomes in Spanish centers to assess the influence of surgical volume and specialized neurosurgical teams on success and complication rates.

**Methods:**

A retrospective, nationwide, study of Spanish centers performing TSS between January 2018 and December 2022. Centers were classified as high volume (HV) [n=11, defined as centers with recognized expertise in Spain or those performing more than 25 TSS/year] or non-HV. Data collection included surgical success rates, complications, and pituitary adenoma resectability (R-PA). Additional analyses evaluated the impact of dedicated neurosurgical teams (DNT) within HV centers.

**Results:**

A total of 2815 TSS from 29 Spanish centers were included (1421 NSPA, 436 GH-secreting, 323 Cushing’s disease, 127 PRL-secreting and 25 TSH-secreting PA). The overall success rate was 50.5%, 76.8% for R-PA. HV centers had a higher overall success rate (53.1 vs. 47.7%; p=0.03). Better TSS outcomes for NSPA accounted for this difference. The overall TSS complication rate was 22.1%, which was higher for NSPA than for SPA (25.0 vs. 17.7%). The overall complication rate of TSS for PA was significantly higher in non-HV centers than in HV centers (24 vs 20.4.0; p <0.01). Centers with a DNT showed a trend to higher success rate in R-PA, while having a lower overall incidence of complications in TSS for PA than HV centers without a DNT (18.5 vs. 23.0; p=0.058), mainly reducing the rate of permanent ADH deficiency in all TSS for PA (2.7 vs. 8.4%; p<0.001).

**Conclusion:**

Higher surgical volume and DNT are associated with improved TSS outcomes for PA in Spain. Our results support the recommendation of concentration of pituitary surgery in a reduced number of centers of expertise in our country in order to improve the success rate and reduce complications, mainly postoperative ADH deficiency.

## 1 Introduction

Transsphenoidal resection is the primary treatment approach for pituitary adenomas (PA) and other tumors near the sella turcica. However, the success rate of surgical procedures varies significantly across studies, as do the associated complication rates. Twenty-five years ago (1), a higher success rate for the surgical treatment of GH-secreting adenomas was reported in patients operated by a single experienced neurosurgeon. Furthermore, these outcomes improved with increasing neurosurgical experience (2). Subsequent studies have corroborated these findings in the context of acromegaly (3, 4) and also in Cushing’s disease (CD) and prolactinomas, with a significantly higher remission rate in centers performing more than ten operations per year for CD (5).

The rate of tumor recurrence at five years after TSS is largely reduced from 44% to 4% in patients with non-secreting pituitary adenomas (NSPA) who have undergone gross total resection (GTR) of the tumor (6). It is increasingly recognized that GTR in NSPA is more frequently achieved through the endoscopic approach compared to the traditional microscopic TSS (7). A study conducted in 2016 demonstrated that even a surgeon with limited experience can achieve comparable outcomes to those of highly experienced surgeon using a microscopic technique, when utilizing endoscopic methods in a cohort of patients with NSPA (8). Moreover, the rate of GTR in NSPA was found to improve with the surgeon’s experience (9).

The complication rate of TSS depends on the annual volume of operations performed by a neurosurgeon and his completion of a learning curve (10). In the last century, some reports suggested a minimum learning curve of 200 operations for microscopic TSS (11) which has been reduced to 40-50 surgeries with the use of endoscopy (12, 13).

The proposal to establish Pituitary Tumors Centers of Excellence (PTCOE) as the ideal model for managing pituitary pathology (14) has received widespread support. Recently, core criteria for the PTCOE accreditation process have been published (15). These criteria include an annual volume of 100 pituitary surgeries per center, mainly TSS. More recently, a large study of 1149 patients in nine PTCOE showed optimal rates for complications of TSS in pituitary adenoma (16).

Despite the growing body of evidence supporting the establishment of PTCOE and the impact of surgical experience on TSS outcomes, there is a lack of comprehensive data on TSS outcomes for PA in Spain. While several countries have published their results in line with PTCOE criteria (17), the situation in Spain remains largely underreported. This absence of data hinders the evaluation of surgical outcomes, the understanding of complication rates, and the potential benefits of centralizing care in specialized centers.

Thus, our aims were to evaluate the rate of successful TSS and the incidence of surgical complications in a large multicenter series in Spain. Considering the influence of surgical experience, case volume and surgical specialization on these outcomes. This will provide a clearer picture of how TSS outcomes align with international benchmarks. Additionally, these findings may serve as a foundation for policy recommendations to optimize pituitary tumor management nationwide.

## 2 Materials and methods

### 2.1 Study design and participants

We evaluated the results of TESSPAIN (TranssphEnoidal Surgery in SPAIN), a retrospective, multicenter, nationwide project that included all the TSS that were performed in the 29 participating centers from January 1, 2018, to December 31, 2022. The study was endorsed by the Spanish Society of Endocrinology and Nutrition (SEEN) and distributed to all members of the SEEN Neuroendocrinology Task Force, which includes most of the endocrinologists who take care of these patients in Spain. The study was reviewed and approved on May 24, 2023, by the Regional Ethics Committee of the Basque Country (CEIm-E) (PI2023077). The study was conducted in accordance with the requirements of the Declaration of Helsinki and good clinical practice. Due to the retrospective nature of the study, patient consent was waived.

For each patient, the coded identity of the neurosurgeon, tumor type, year of surgery, therapeutic goal and outcome, and postoperative complications were recorded.

Six types of lesions were identified: Clinically non-secreting pituitary adenoma (NSPA); Growth hormone-secreting pituitary adenoma, including those cosecreting prolactin or other hormones (ACRO); Cushing’s disease (CD); prolactin-secreting adenoma (PRLoma); thyroid-stimulating hormone-secreting adenoma (TSHoma); and other non-adenomatous tumors, mainly craniopharingiomas, meningiomas, chordomas, and Rathke’s cleft cysts (OTHER). ACRO, CD, PRLoma, and TSHoma were evaluated globally as Secreting Pituitary Adenoma (SPA).

Centers that were accredited in Spain during the study period (referred to as CSUR centers in Spain) and those performing more than 25 surgeries per year were classified as high-volume (HV) centers. These HV centers were compared with those not meeting these criteria, referred to as non-high-volume (non-HV) centers.

In Spain, CSUR centers meet several specific requirements (see footnote^1^), including performing more than 20 pituitary surgeries annually over the past three years, having a multidisciplinary team, managing more than 250 patients with pituitary diseases regularly attended, and conducting teaching and research activity, among others.

HV centers with a dedicated neurosurgeon who performed more than 75% of all TSS or with a stable team of up to 3 dedicated neurosurgeons were compared with HV centers without a dedicated neurosurgeon or without an established team.

### 2.2 Definitions and objectives

Surgical success for SPA was defined according to the published remission criteria for each disease: age normalized serum IGF-1 value and a random GH <1.0 µg/L for ACRO (18); postoperative basal cortisol less than 5 µg/dl and adrenal insufficiency requiring steroid replacement for more than 3 months for CD (19); serum prolactin less than 10 ng/ml for PRLoma, recently reported as associated with low recurrence (20), and resolution of hyperthyroidism for TSHoma. For NSPA, surgical success was considered when GTR was confirmed on the MRI three to six months after TSS. At each participating center, the assessment of GTR for NSPA was performed locally. MRIs were reviewed by neuroradiology teams as part of routine follow-up at three to six months postoperatively. These teams included experienced neuroradiologists with expertise in pituitary imaging. While MRI review was not centralized, all centers adhered to established criteria for reporting residual tumor to ensure consistency of assessment (21).

The following surgical complications were evaluated: postoperative cerebrospinal fluid (CSF) leak, reoperation for bleeding or CSF leak, infection, permanent anterior pituitary hormone or arginine vasopressin deficiency (AVPD) persisting beyond 6 months of surgery, venous thromboembolism, cerebrovascular accident, cranial oculomotor nerve or optic nerve injury, death, and other (including pneumocephalus and vasospasm). New anterior pituitary deficiencies were based on the prescription of new hormonal replacement therapy after surgery, without specifying either the number of axes or which ones.

Both outcomes, surgical success and surgical complications, were evaluated based on whether the hospital met or not the HV criteria. For HV centers, the impact of having a dedicated neurosurgical team (DNT) on these outcomes was also evaluated.

In addition, adenomas retrospectively deemed resectable (R-PA) were evaluated for surgical success and complications. To qualify as resectable, those PA with preoperative advanced cavernous sinus invasion (Knosp 3b or 4) documented in the radiologic report or after the revision of the preoperative MRI (if not previously specified), were excluded.

### 2.3 Statistical analyses

Categorical variables were expressed as absolute values (n) and percentages (%), while quantitative data were presented as mean ± standard deviation or median and interquartile range for non-normally distributed parameters. Success rate and complication rate were the main outcomes. Outcomes were calculated as percentages with total numbers and reported with 95% confidence intervals calculated using data weighted by the number of cases operated in each center. Outcomes were compared using the chi-squared test or the Fisher´s exact test to evaluate the influence of being treated in an HV center versus not being treated in an HV center on both success and complication rates, and to assess the effect of having a dedicated neurosurgical team between HV centers with and without a dedicated neurosurgical team. Spearman’s coefficient (Rho) was calculated to describe the correlation between total TSS and number of TSS for PA at each center with success rate. The correlation between surgical volume and complication rates at each center and the correlation between success rates and complication rates at each center were also evaluated using this coefficient. Statistical analyses were performed using IBM SPSS Statistics version 27.0.

## 3 Results

### 3.1 Population study

**Figure 1:**
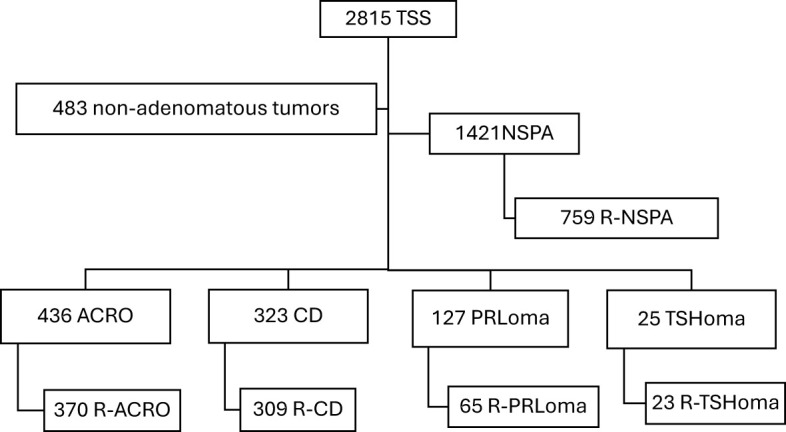
Flowchart of the patients included in the TESSPAIN Registry.

A total of 2815 TSS procedures were performed at the 29 study centers. The most frequent PA type was NSPA (n=1421; 50.5%), followed by SPA (n=911; 32.4%): 436 patients with ACRO, 323 patients with CD, 127 patients with PRLoma, and 25 operated for TSHoma. The remaining 483 TSS (17.1%) were performed for non-adenomatous pituitary tumors, usually with extrasellar extension, mainly for craniopharyngioma, Rathke´s cleft cyst, meningioma, chordoma, and others (**Figure 1**). More than 80% of SPAs were considered R-PA by the referring endocrinologists, with the exception of PRLomas where only 56.7% were considered resectable.

Nine centers had the recognition as CSUR, and these, along with two others performing more than 25 TSS/year formed the HV group. This group had performed a higher percentage of TSS on non-adenomatous tumors and R-NSPA than the non-HV group, and HV group also had a lower percentage of R-ACRO than non-HV group. **Table 1** shows the details of the included cases and their comparison in both groups.

**Table 1:** Number of cases and comparison divided by the expertise of the operating center.

	Total (%)	Mean/median*	Range	HV centers	Non-HV centers	p value^$^
TSS	2815 (100)	97.1 ± 41.5	24-180	1505	1310	**-**
PA (% of TSS)	2332 (82.4)	80.4 ± 31.5	24-130	1212 (80.5)	1120 (85.5)	<0.01
R-PA (% of PA)	1534 (65.8)	68.7 ± 12.1	7-100	816 (67.3)	718 (64.1)	0.10
Other tumors	483 (17.6)	14 (17)	0-58	293 (19.5)	190 (14.5)	<0.01
SPA (% of PA)	911 (39.1)	39.1 ± 8.3	10-59	493 (40.7)	418 (37.3)	0.10
R-SPA (% of SPA)	774 (85)	87.2 ± 8.1	4-47	409 (83.0)	365 (87.2)	0.07
ACRO (% of PA)	436 (18.7)	15.0 ± 6.2	3-26	226 (18.6)	210 (18.7)	0.95
R-ACRO (% of ACRO)	370 (84.9)	18 (2)	9-19	182 (80.5)	188 (89.5)	<0.01
CD (% of PA)	323 (13.8)	11.1 ± 7.0	1-25	184 (15.2)	139 (12.4)	0.05
R-CD (% of CD)	309 (95.7)	10.7 ± 7.0	4-25	173 (94.0)	136 (97.8)	0.09
PRLoma (% of PA)	127 (5.4%)	4.4 ± 3.2	0-12	69 (5.7)	58 (5.2)	0.58
R-PRLoma (% of PRLoma)	72 (56.7)	4 (7)	0-11	42 (60.9)	30 (51.7)	0.30
TSHoma (% of PA)	25 (1.1)	0 (2)	0-5	14 (1.2)	11 (1.0)	0.68
R-TSHoma (% of TSHoma)	22 (92)	2 (5)	0-5	12 (85.7)	11 (100)	0.30
NSPA (% of PA)	1421 (60.9)	49 ± 19.7	13-87	719 (59.3)	702 (62.7)	0.10
R-NSPA (% of NSPA)	760 (53.5)	26.2 ± 17.5	0-66	407 (56.6)	353 (50.3)	0.01

TSS, transsphenoidal surgery; PA, pituitary adenoma; ACRO, GH, secreting adenomas; CD, Cushing´s disease; PRLoma, prolactin secreting adenoma; TSHoma, TSH secreting adenoma; SPA, secreting PA; NSPA, non-secreting pituitary adenoma; R-PA, resectable PA; HV, High Volume; R-, resectable.

*Mean or median per center, depending on the distribution. ^$^Between HV centers and non-HV centers.

Six of the eleven HV centers had a dedicated neurosurgical team, while five of them had more than three neurosurgeons, and none of them performed more than 75% of the TSS.

### 3.2 Success rate of TSS

The overall success rate of TSS for PA was 50.5%, increasing to 76.8% for R-PA. TSS success for each PA subtype increased when considered for R-PA separately, particularly for PRLoma and NSPA. TSH-secreting adenomas had the highest success rate, 88% (CI_95:_ 75.7-100%), increasing to 95.6% for R-TSHoma. These results are shown in **Table 2**.

**Table 2:** Success rates (95% CI) of transphenoidal surgery for PA.

	OVERALL	HV center (n:11)	Non-HV center (n:17)	p
Number of patients	2332	1212	1120	**-**
PA	50.5 (49.9-51.0)	53.1 (52.4-53.9)	47.7 (46.8-48.5)	0.03
R-PA (n= 816 vs. 718)	76.8 (76.2-77.4)	78.9 (78.4-79.5)	74.4 (73.3-75.5)	0.03
SPA (n= 493 vs. 418)	61.2 (60.4-62.1)	60.0 (59.2-60.9)	63.4 (61.9-64.9)	0.30
R-SPA (n= 409 vs. 365)	72.7 (71.9-73.6)	72.4 (71.3-73.4)	72.6 (71.1-74.1)	0.94
ACRO (n= 226 vs. 210)	58.5 (57.1-59.8)	55.3 (54.2-56.4)	61.9 (59.4-64.4)	0.16
R-ACRO (n= 182 vs. 188)	68.9 (67.3-70.5)	68.7 (66.9-70.5)	69.1 (66.5-71.7)	0.92
CD (n= 184 vs. 139)	71.5 (69.4-73.6)	69.6 (66.8-72.4)	74.1 (70.9-77.3)	0.37
R-CD (n= 173 vs. 136)	74.8 (72.9-76.6)	74.0 (71.7-76.3)	75.7 (72.60-78.9)	0.73
PRLoma (n= 69 vs. 58)	41.7 (36.3-47.1)	46.4 (38.7-54.1)	36.2 (28.9-43.4)	0.25
R-PRLoma (n= 42 vs. 30)	73.6 (67.7-79.5)	76.2 (69.3-83.0)	70.0 (59.3-80.7)	0.56
NSPA (n= 719 vs. 702)	43.8 (42.6-45.0)	48.4 (46.8-50.0)	38.3 (36.6-40.0)	<0.01
R-NSPA (n= 407 vs. 353)	81.2 (80.0-82.4)	85.5 (84.5-86.5)	76.2 (74.1-78.3)	<0.01

PA, pituitary adenoma; SPA, secreting pituitary adenoma; ACRO, GH secreting adenomas; CD, Cushing´s disease; PRLoma, prolactin secreting adenoma; TSHoma, TSH secreting adenoma. NSPA, non-secreting pituitary adenoma; R-PA, resectable PA.

TSHomas were excluded due to the low number of cases. Comparison between high-volume (HV) centers and non-high-volume centers (non-HV).

The overall success rate of TSS for PA was significantly higher in HV centers than in non-HV centers (53.1 vs 47.7%; p=0.03). Better outcomes with TSS for NSPA accounted for this difference. This difference persisted when including only R-PA, despite a higher percentage of NSPA considered amenable to GTR in HV centers (**Table 1** **).** The difference in SPA did not reach statistical significance for any subtype, either overall or for R-PA (**Table 2**).

**Figure 2:**
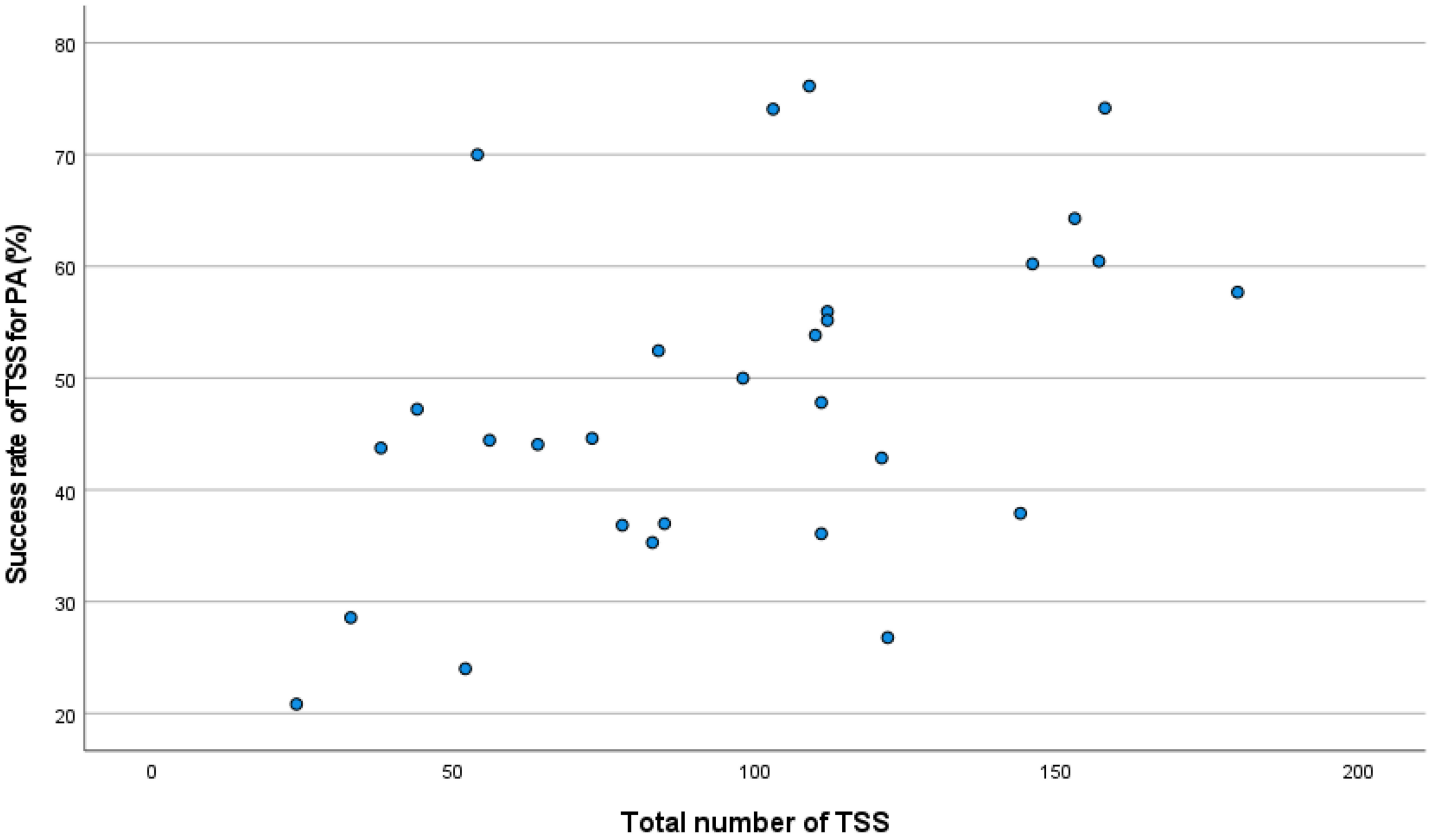
Correlation between total TSS and success rate in PA.

Linear correlation analysis showed a positive correlation between the global success rate in PA and the number of TSS procedures at each center (Rho: 0.49; p <0.01; **Figure 2**), mainly driven by this correlation in R-NSPA (Rho: 0.57; p: <0.01). The success rate in SPA showed only a weak positive correlation with the number of TSS for PRLoma (Rho: 0.38; p: 0.04).

### 3.3 Complications of TSS

The overall surgical complication rate of TSS for all PA was 22.1%, which was higher for NSPA compared to SPA (25.0% vs. 17.7%; p<0.001). The complication rate ranged from 0 to 42.9% in R-SPA. Only one of the five centers with no complications performed more than 10 TSS procedures, corresponding to a HV center with a DNT. The complication rate in R-NSPA was 0% in only one center, which performed 12 cases during the study period. Persistent AVPD occurred in 5.4%, CSF leak in 3.1% and reoperation for complications in 2.8% of all TSS for PA. For SPA, the mean complication rate for R-SPA was not significantly different from the mean complication rate for non-resectable adenomas (18.5% vs. 13.1%; p: 0.13), while this rate was lower for TSS for ACRO than for TSS for CD (13.1% vs. 24.1%; p: 0.01), even when only R-PA were considered (13.5% vs. 24.3%; p<0.001). Complications were more common in TSS for NSPA in non-resectable adenomas compared to R-NSPA (28.1% vs. 22.2% p: 0.01). The mean rate of damage to anterior pituitary function was 14.7% but showed a wide range between centers from 0 to 37.1%. There was a positive correlation between the rate of postoperative anterior pituitary deficiencies for NSPA and the rate of NSPA deemed resectable (Rho: 0.386; p<0.001).

The overall complication rate of TSS for PA was significantly higher in non-HV centers than in HV centers (24.0% vs 20.4%; p<0.01). The difference did not reach statistical significance for any subtype of PA, neither overall nor for R-PA. Ten out of the 2332 patients who underwent TSS for PA died in the postoperative period (surgical mortality for adenomas: 0.4%). The mortality rate was significantly higher in non-HV centers than in HV centers, but with a small number of cases (2 deaths vs. 8 deaths). These results are presented in **Table 3**.

**Table 3:** Global complication rates (95% CI) of transsphenoidal surgery for PA and comparison between high-volume (HV) centers and non-high-volume centers (non-HV).

	OVERALL*	Range	HV center (n:11)	Non-HV center (n:17)	p
PA (n= 1212 vs. 1120)	22.1 (21.6-22.6)	6.8-46.3	20.4 (19.6-21.1)	24.0 (23.3-24.7)	<0.01
R-PA (n= 816 vs. 718)	20.3 (19.7-21.0)	3.2-47.1	18.9 (18.0-19.7)	22.0 (21.1-22.9)	0.13
SPA (n= 493 vs. 418)	17.7 (16.9-18.4)	2.0-41.2	16.2 (15.2-17.2)	19.4 (18.3-20.4)	0.21
R-SPA (n= 409 vs. 365)	18.5 (17.7-19.4)	0.0-42.9	17.6 (16.5-18.7)	19.5 (18.1-20.9)	0.51
ACRO (n= 226 vs. 210)	13.1 (12.0-14.1)	0.0-44.4	13.3 (11.9-14.6)	12.9 (11.2-14.5)	0.90
R-ACRO (n= 182 vs. 188)	13.5 (12.3-14.7)	0.0-40.0	14.8 (13.2-16.5)	12.2 (10.5-14.0)	0.46
CD (n= 184 vs. 139)	24.1 (21.9-26.4)	0.0-62.5	21.2 (18.4-24.0)	28.1 (24.5-31.6)	0.15
R- CD (n= 173 vs. 136)	24.3 (22.0-26.5)	0.0-62.5	21.4 (18.5-24.3)	27.9 (24.4-31.5)	0.18
PRLoma (n= 69 vs. 58)	17.3 (13.6-22.2)	–	14.5 (9.1-19.8)	20.7 (14.6-26.8)	0.36
R-PRLoma (n= 42 vs. 30)	15.3 (10.8-19.9)	–	16.7 (8.2-25.1)	23.3(14.4-32.5)	0.48
NSPA (n= 719 vs. 702)	25.0 (24.2-25.8)	7.3-53.8	23.2 (22.1-24.4)	26.8 (25.7-27.9)	0.12
R-NSPA (n= 407 vs. 353)	22.2 (21.8-22.9)	0.0-42.9	20.1 (18.8-21.4)	24.6 (23.1-26.2)	0.13
Permanent AVPD	5.4 (5.2-5.7)	0.0-24.1	5.0 (4.7-5.4)	5.9 (5.6-6.2)	0.36
Permanent AVPD NSPA	6.4 (6.0-6.8)	0.0-38.5	6.3 (5.8-6.8)	6.0 (5.5-6.5)	0.83
Reintervention	2.8 (2.7-2.9)	0.0-6.2	2.4 (2.3-2.5)	3.1 (3.0-3.3)	0.28
Reintervention NSPA	3.5 (3.3-3.7)	0.0-14.3	2.8 (2.6-3.0)	4.1 (3.8-4.4)	0.16
CSF leak	3.1 (3.0-3.2)	0.0-9.9	2.7 (2.6-2.8)	3.5 (3.3-3.7)	0.41
CSF leak NSPA	3.2 (3.0-3.4)	0.0-15.6	2.6 (2.5-2.8)	3.6 (4.3-3.6)	0.16
APD	14.7 (14.2-15.3)	0.0-37.1	14.4 (13.7-15.2)	15.1 (14.4-15.8)	0.36
APD NSPA	16.7 (16.0-17.5)	0.0-43.1	17.0 (15.8-18.1)	16.8 (15.7-17.9)	0.77
Mortality	0.43 (0.36-0.50)	0.0-2.6	0.17 (0.15-0.19)	0.71 (0.66-0.76)	0.04

*Including only centers with more than five TSS in these categories. Data for TSHoma re not shown.

PA, pituitary adenoma; SPA, secreting pituitary adenoma; ACRO, GH secreting adenomas; CD, Cushing´s disease; PRLoma, prolactin secreting adenoma; TSHoma, TSH secreting adenoma. NSPA, non-secreting pituitary adenoma; R-PA, resectable PA; AVPD, arginine-vassopresin deficiency; CSF, cerebrospinal fluid; APD, anterior pituitary deficiency.

Linear correlation analysis showed no positive or negative correlation between the overall complication or AVPD rate in PA and the number of TSS procedures in each center. Neither the total number of TSS nor the number of TSS for PA in each center had any correlation with the complication rate in all groups (divided by tumor type or complication type). However, a correlation was found between the global success rate in PA and the complication rate in R-PA (Rho: 0.499; p: 0.006). This positive correlation with global success rate remained significant when R-SPA (Rho: 0.454; p: 0.006) and R-NSPA (Rho: 0.488; p: 0.008) were evaluated separately.

### 3.4 Influence of a dedicated neurosurgical team in HV centers

The six HV centers with a DNT operated on 712 pituitary adenomas, of which 60.9% were NSPA (n: 434) and 48.4% (n: 210) were considered to be R-NSPA. The five HV centers without a DNT operated 500 pituitary adenomas, of which 57% were NSPA (n: 285) and 69.1% (n: 197) R-NSPA, significantly higher rate than in centers with a DNT (p<0.001). The success rate for PA was significantly higher in centers without a DNT due to a higher rate of GTR in NSPA. However, this difference disappeared when only R-NPSA were included. Success rate was higher without statistical significance for all SPA types except PRLoma, with greater difference for R-SPA. These data are shown in **Table 4**.

**Table 4:** Comparison of success rates and complications rates between patients operated in a HV center with and without a dedicated neurosurgical team.

SUCCESS RATE	DNT % (95% CI) (n:6)	Not DNT % (95% CI) (n:5)	p
Number of patients (n=1212)	712	500	–
PA	49.7 (48.5-50.9)	58.0 (57.6-58.3)	<0.01
R-PA (n= 438 vs. 378)	80.8 (80.0-81.6)	76.7 (76.0-77.4)	0.15
SPA (n= 278 vs. 215)	62.2 (60.9-63.6)	57.2 (56.3-58.1)	0.26
R-SPA (n= 228 vs. 181)	75.9 (74.9-76.6)	68.0 (66.2-69.7)	0.07
ACRO (n= 118 vs. 108)	60.2 (59.3-61.0)	50.0 (48.3-51.7)	0.12
R-ACRO (n= 96 vs. 86)	74.0 (72.9-75.0)	62.8 (59.5-66.0)	0.10
CD (n= 110 vs. 74)	70.9 (67.1-74.7)	67.6 (63.5-71.6)	0.63
R- CD (n= 104 vs. 69)	75.0 (71.8-78.2)	72.5 (69.2-75.7)	0.71
PRL (n= 40 vs. 29)	40.0 (29.2-50.8)	55.2 (44.5-65.8)	0.21
R-PRL (n= 20 vs. 22)	80.0 (74.8-85.2)	77.5 (60.1-85.3)	0.72
NSPA (n= 434 vs. 285)	41.7 (39.4-44.0)	58.6 (57.4-59.8)	<0.01
R-NSPA (n= 210 vs. 197)	86.2 (84.5-88.0)	84.8 (83.9-85.6)	0.68
COMPLICATIONS RATE	DNT % (95% CI) (n:6)	Not DNT % (95% CI) (n:5)	p
PA	18.5 (17.5-19.5)	23.0 (21.9-24.1)	0.06
R-PA (n= 438 vs. 378)	16.2 (15.3-17.1)	22.0 (20.5-23.4)	0.04
SPA (n= 278 vs. 215)	14.7 (13.5-16.0)	18.1 (16.5-19.8)	0.31
R-SPA (n= 228 vs. 181)	15.9 (14.7-17.1)	19.8 (17.9-21.8)	0.41
ACRO (n= 118 vs. 108)	11.0 (9.4-12.6)	15.7 (13.5-17.9)	0.30
R-ACRO (n= 96 vs. 86)	11.5 (9.5-13.4)	18.6 (16.0-21.2)	0.18
CD (n= 110 vs. 74)	20.9 (16.5-25.3)	21.6 (19.2-24.0)	0.91
R- CD (n= 104 vs. 69)	22.1 (17.5-26.7)	20.3 (17.9-22.7)	0.77
PRL (n= 40 vs. 29)	10.0 (5.3-14.7)	20.7 (9.7-31.7)	0.21
R-PRL (n= 20 vs. 22)	10.0 (2.7-17.3)	22.7 (7.8-37.6)	0.41
NSPA (n= 434 vs. 285)	21.0 (19.4-22.5)	26.7 (25.1-28.3)	0.08
R-NSPA (n= 210 vs. 197)	16.2 (14.9-17.5)	24.4 (22.2-26.5)	0.04
Permanent AVPD	2.7 (2.6-2.8)	8.4 (7.6-9.2)	<0.01
CSF leak	2.4 (2.2-2.6)	3.2 (3.1-3.3)	0.39
Reintervention	2.1 (1.9-2.3)	2.8 (2.6-3.0)	0.44
Anterior pituitary deficiency	13.3 (12.4-14.3)	16.0 (14.8-17.2)	0.09

Data for TSHoma are not shown.

DNT, dedicated neurosurgical team; PA, pituitary adenoma; SPA, secreting pituitary adenoma; ACRO, GH secreting adenomas; CD, Cushing´s disease; PRLoma, prolactin secreting adenoma; TSHoma, TSH secreting adenoma; NSPA, non-secreting pituitary adenoma; R-PA, resectable PA; AVPD, arginine-vassopresin deficiency; CSF, cerebrospinal fluid.

Complication rates were lower in R-PA operated at HV centers with a DNT (16.2 vs. 22.0; p=0.04) due to less surgical complications in R-NSPA, with no differences in secreting adenomas. The rate of permanent AVPD (2.7 vs. 8.4%) was the most significant difference. These data are also shown in **Table 4**.

## 4 Discussion

The outcomes of transsphenoidal surgery (TSS) are mostly influenced by tumor characteristics, -particularly its size and invasiveness-, as well as the expertise and experience of the medical team performing the surgery. Higher success rates with increasing neurosurgical experience have been reported for the surgical treatment of secreting adenomas, particularly in patients operated on by a single experienced neurosurgeon (2–5). The same has been demonstrated for TSS for NSPA, with improved outcomes and a higher rate of GTR after completing a learning curve (9). Previously reported series refer to high expertise centers performing high volume TSS (12), but there is a lack of data including daily practice in a nationwide setting like TESSPAIN.

The TESSPAIN study provides a comprehensive evaluation of TSS outcomes for pituitary PA across 29 centers in Spain. This study’s findings offer valuable insights into the effects of surgical experience, case volume, and specialized neurosurgical teams on surgical success and complication rates in treating PA, contributing essential data to the ongoing discussions about PTCOE. The overall success rate of TSS was higher in centers with HV with lower complications. The annual number of TSS recommended to reduce the risk of complications from TSS was set at 25 transsphenoidal operations for PA per year in the review by Honegger et al. (5), and this number was selected to classify a center as HV, in addition to nationally recognized centers of excellence for pituitary surgery.

### 4.1 Comparison of high- and low-volume centers

Our results show that compared to non-HV centers, HV centers with greater experience achieved a higher overall success rate for PA surgery. This association between surgical volume and outcomes aligns with international literature (5, 9), which supports the importance of high case volumes in improving surgical success. Specifically, higher success rates were particularly notable in NSPA in HV centers, where the ability to achieve GTR plays a critical role. Moreover, a significant positive correlation was observed between the global success rate and the number of TSS procedures per center, reinforcing the impact of experience and surgical practice volume on clinical outcomes.

HV centers demonstrated a lower overall complication rate, highlighting the safety advantages associated with increased surgical volume. However, this trend did not consistently reach statistical significance across all PA subtypes, possibly due to variations in adenoma characteristics and surgical approaches across different centers. These findings support the need for PTCOE designation, where volume thresholds can help standardize care quality.

The lack of a significant difference in the overall success rate for each type of secreting adenoma between centers with and without a dedicated neurosurgical team may be explained by the limited number of operations performed in each center included in the study.

### 4.2 Impact of a dedicated neurosurgical team

In HV centers, having a DNT was associated with notable benefits, especially regarding postoperative outcomes for NSPA. Centers with a DNT showed lower complication rates, particularly in persistent AVPD, with a rate similar to the 2% (95% CI, 0.02-0.03) recently reported in a systematic review of the literature (22). The presence of a stable, specialized team may contribute to refined procedural skills, enabling safer resections and quicker management of complications.

Interestingly, while HV centers with a DNT showed significantly fewer complications in R-NSPA, the success rate for all NSPA was higher in HV centers without a DNT. This difference disappeared when only R-NSPA were considered. This fact may reflect case selection dynamics, as HV centers without a DNT handled a lower proportion of complex cases attempting to achieve GTR (48.4% vs. 69.1%) and performed more TSS without curative intention. Regardless, the lower complication rates observed in centers with a DNT highlight the potential value of such teams in optimizing surgical safety.

### 4.3 Surgical Complications and Influencing Factors

The overall complication rate in this study was 22.1%, with new anterior pituitary deficiencies being the most common postoperative morbidity, followed by persistent AVPD. Although there was no significant difference in AVPD rates between HV and non-HV centers, the incidence was notably lower when surgeries were performed by a dedicated DNT. This finding suggests that although AVPD remains a risk, its incidence may be mitigated by increased surgical experience and procedural improvements. A positive correlation was noted between the rate of postoperative anterior pituitary deficiency and the percentage of NSPA deemed resectable. In addition, the lower rate of NSPA amenable to GTR in non-HV centers suggests that these centers usually operated on more invasive NSPA with worse preoperative anterior pituitary function that could not be impaired by TSS, often performed without curative intention. Dedicated neurosurgeons in HV centers achieved the same success rate in R-NSPA with significantly fewer complications than non-dedicated neurosurgeons in HV centers, as reported many years ago (11).

The mortality rate for TSS was low at 0.4%, in line with international standards (23). However, the mortality rate was higher in non-HV centers, which may be due to the differences in experience in the management of complex cases. This finding highlights the importance of specialized and experienced surgical teams in minimizing risks associated with PA surgeries.

### 4.4 Outcomes by tumor type

Longitudinal studies have shown that the success rate of TSS in acromegaly continues to improve over decades, especially with increasing experience (24, 25) and a dedicated neurosurgeon (3, 26). With data collected from nine PTCOE, the remission rate for TSS in ACRO with macroadenomas was 49% without taking into account the invasiveness of the tumor (15). The remission rate in noninvasive adenomas was 62.9% in another study that showed an improvement rate with increasing experience, 50 to 73.6% in two periods (24). Our success rate was similar, 68.9% for R-ACRO. This rate improved with DNT in HV centers (74%), but did not reach statistical significance, probably because of the small number of cases in each center. However, the success rate for R-ACRO was similar, with no difference between HV and non-HV centers.

Two recent studies reported a global remission rate of 72.5% and 88.1% in TSS for CD (27, 28), slightly higher than ours (71.5%). We found a non-significant higher success rate in HV centers with DNT for CD and R-CD, and there was no significant difference in success rate when comparing HV centers with non-HV centers. This finding may be partially explained by the lower rate of R-CD in HV centers.

Invasion of the cavernous sinus by PRLoma is a poor prognostic factor (29) for successful TSS. Almost half of our PRLomas (43.3%) were not resectable. In a review published in 2020 (30), long-term disease control after surgery was 67% (95% CI, 60-74), 83% for microprolactinomas and 60% for macroprolactinomas. HV centers with a dedicated neurosurgical team achieved a success rate of 80% for R-PRLomas, but the small number of patients precluded evaluation of these results.

An earlier diagnosis of TSHomas allows more microadenomas to be identified, as in our case with a resectable rate of 92% for TSHomas and a success rate of 88%, which is much higher than that published in older series (31).

The ideal outcome of TSS for NSPA should be GTR, as regrowth of residual tumor can occur in up to 50% of cases (6). Invasiveness is the main determinant of the likelihood of GTR. In their review of 28 previous publications, Honegger et al. found no significant correlation between GTR and the annual number of TSS cases (5). HV centers had higher GTR than non-HV centers in our series. Similarly, there was a positive correlation between the total number of TSS and GTR for both NSPA and R-NSPA. This correlation of GTR rate has less significance with the number of TSS for PA at each center, suggesting that performing TSS for other tumors may predict better outcomes in NSPA. In addition, endocrinologists from HV centers were more likely to consider NSPA as amenable to GTR, suggesting a more demanding attitude towards GTR for NSPA in centers with a higher number of TSS. A DNT did not significantly increase the rate of GTR for R-NSPA in HV centers (86.2 vs. 84.8%), although they had fewer surgical complications for R-NSPA. The 75th percentile cut-off for residual tumor in the benchmark outcomes study for transsphenoidal surgery of pituitary adenomas for R-NSPA was less than 23.2% (16). In our study, the GTR rate for R-NSPA was 81.2%, which is better. However, this value may be influenced by the criteria used by each endocrinologist to consider an NSPA as resectable.

### 4.5 Study implications and future directions

The TESSPAIN study bring out the importance of establishing PTCOE standards in Spain, as this could enhance consistency in TSS outcomes across centers. By concentrating resources and expertise in designated centers, Spain could improve surgical success rates and minimize complications associated with PA surgery. This model could particularly benefit patients in non-HV centers, where access to specialized teams and high case volumes remains limited.

Additionally, the correlation between surgical success and volume supports the necessity of experience for optimal outcomes, reinforcing the value of implementing volume thresholds in accreditation criteria.

### 4.6 Limitations

The retrospective design of this study and the reliance on self-reported data from multiple centers may introduce reporting biases or inconsistencies in data collection methods. This is particularly true for the assessment of resectability, which was based on radiologic report or review of images at each center, and the detection of new postoperative hormonal deficiencies, which was based on the prescription of new hormonal replacement therapy after surgery without detailed data. Furthermore, while surgical success rates and complications were correlated with volume and specialization, causal inferences are limited. Prospective studies with standardized data collection protocols are warranted to validate these findings.

The main strengths of this study are the large number of operations and the wide range of centers reflecting the real-world experience in Spain.

### 4.7 Conclusion

The TESSPAIN study provides essential data on TSS outcomes for PA in Spain, revealing that surgical experience, high case volumes, and specialized neurosurgical teams are instrumental in achieving optimal surgical success and minimizing complications. These findings support the establishment of PTCOE in Spain to ensure that patients with PA receive the highest quality of care. With standardized benchmarks and focused expertise, PTCOE could reduce variability in outcomes, aligning Spain’s care standards with international best practices for managing pituitary adenomas.

## Data Availability

The raw data supporting the conclusions of this article will be made available by the authors, without undue reservation.

## Footnotes

1 Ministerio de Sanidad.
https://www.sanidad.gob.es/profesionales/CentrosDeReferencia/CentrosCSUR.htm [Accessed September 10, 2024]
